# A Glimpse at Siderophores Production by *Anabaena flos-aquae* UTEX 1444

**DOI:** 10.3390/md20040256

**Published:** 2022-04-06

**Authors:** Roberta Teta, Germana Esposito, Karishma Kundu, Mariano Stornaiuolo, Silvia Scarpato, Antonino Pollio, Valeria Costantino

**Affiliations:** 1 “TheBlueChemistryLab”, Department of Pharmacy, Task Force “BIGFED2”, University of Naples Federico II, Via Domenico Montesano 49, 80131 Napoli, Italy; roberta.teta@unina.it (R.T.); germana.esposito@unina.it (G.E.); karishma.kundu@unina.it (K.K.); silvia.scarpato@unina.it (S.S.); 2 Department of Pharmacy, University of Naples Federico II, Via Domenico Montesano 49, 80131 Napoli, Italy; mariano.stornaiuolo@unina.it; 3 Department of Biology, Complesso Universitario Monte Sant’Angelo via Cinthia–Edificio 7, University of Naples Federico II, 80126 Napoli, Italy; antonino.pollio@unina.it

**Keywords:** siderophores, cyanobacteria, iron, molecular networking, *Anabaena flos-aquae*, hydroxamates, catecholates, carboxylates, schizokinen, synechobactins, natural products, biodiversity

## Abstract

In this study, a strain of *Anabaena flos-aquae* UTEX 1444 was cultivated in six different concentrations of iron (III). Cultures were extracted with organic solvents and analyzed using our dereplication strategy, based on the combined use of high-resolution tandem mass spectrometry and molecular networking. The analysis showed the presence of the siderophores’ family, named synechobactins, only in the zero iron (III) treatment culture. Seven unknown synechobactin variants were present in the extract, and their structures have been determined by a careful HRMS/MS analysis. This study unveils the capability of *Anabaena flos-aquae* UTEX 1444 to produce a large array of siderophores and may be a suitable model organism for a sustainable scale-up exploitation of such bioactive molecules, for the bioremediation of contaminated ecosystems, as well as in drug discovery.

## 1. Introduction

Living organisms require several kinds of metals for their wellness. In virtue of its unique coordination and redox chemistry, iron is, among the metal ions, involved in vital metabolic functions in plants, animals, and microorganisms. The two most frequent oxidation states of iron are +2 and +3, which are also referred to as ferrous and ferric, respectively. Because of its oxidation states, iron plays crucial roles in biocatalysis and electron transport chains, in several biological systems [1]. Microbes use different methods to collect iron from the environment; among others, they produce Fe^3+^-chelating molecules, named siderophores [2]. Siderophores are low-molecular-weight molecules (500–1500 Da), endowed with and named after their high affinity for iron (III) (Kf > 1030). Pathogens adapt their own iron-uptake strategy, in response to the type of infection (acute or chronic) and to the availability of iron [3] of the host. Acting as ferric ion scavengers, siderophores contribute significantly to the virulence of pathogenic microbes. Fe^3+^ is transported inside the cell as a hexadentate octahedral complex with the siderophores. Depending on the primary oxygen-donating ligands that bind the iron, siderophores are classified as follows: hydroxamates, catecholates, carboxylates, and mixed-types siderophores [4].

Siderophores are powerful molecules that have applications in ecological research; for the removal of petroleum hydrocarbons from oceans and as algal bloom biocontrollers, in agriculture as soil bioremediators, and in drug discovery as iron chelation therapy, antibiotic carriers, and fish pathogen biocontrollers [5,6,7]. The most intriguing application is their use as ligands for antibiotics, the so-called “Trojan horse” strategy [8]. The potential chemical space of siderophore–antibiotic conjugates is a great opportunity to explore an untapped natural resource and design a new class of molecules to face drug-resistant pathogens [6,9]. 

**Figure 1:**
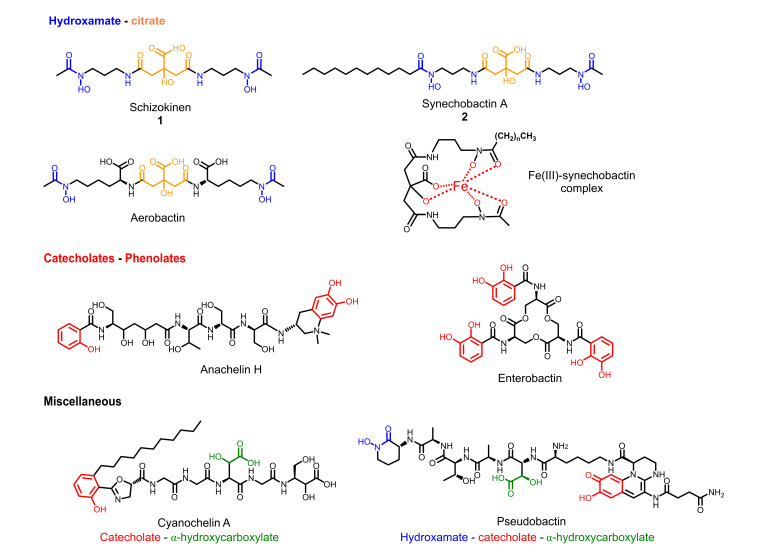
Examples of cyanobacterial siderophores. Iron-binding moieties are highlighted as follows: hydroxamate-citrate units are colored in blue-orange, catecholates-phenolates are in red, α-hydroxy carboxylate is in green.

Cyanobacteria are a class of photosynthetic microorganism, spread out in a large array of environments, from tropical areas to extremely cold waters. They are present in marine waters, as well as in freshwaters. This unique class of microorganisms is well known to produce several different classes of secondary metabolites, either toxins, named cyanotoxins, or bioactive natural products with interesting pharmacological properties [10]. Cyanobacteria rely on ferric iron to survive. While iron is one of the most abundant elements on Earth, bioavailable iron in freshwater and marine environments is limited, falling in the picomolar to a low nanomolar range. To survive in such an iron-depleted environment, cyanobacteria produce siderophores. Recently, it has been further speculated that cyanobacteria use siderophores as antimicrobial agents and as shields, to protect themselves from heavy-metal toxicity [11]. Cyanobacterial siderophores (Figure 1) include mainly either hydroxamates or catecholates [12]. The first evidence of presence and types of siderophores in cyanobacteria date back to the early 1980s, when iron uptake in *Anabaena* sp. in iron starvation conditions was demonstrated to be mediated by the hydroxamate-type siderophore schizokinen [13].

On the other hand, cyanobacterial genomes are known to harbor a rich variety of gene clusters with unknown function. Recently, we reported on the awakening of a widely distributed class of silent gene clusters by iron starvation that yielded cyanochelin’s production, β-hydroxy aspartate lipopeptides involved in iron uptake [14].

It is well known that a single strain produces different molecules when grown under different environmental conditions. This concept is the basis of the OSMAC (“One Strain, Many Compounds”) strategy [15], a cultivation-based approach that consists in altering cultivation parameters, such as nutrient content, metal ions, rate of aeration, temperature, in order to trigger the productions of compounds of biomedical interest and to activate silent biosynthetic gene clusters. 

In this context, in the frame of our ongoing research on the potentiality of cyanobacteria as incubators of bioactive molecules, we investigated the metabolome of the strain of *Anabaena flos-aquae* (UTEX 1444), when cultivated in the condition of iron deficiency and iron overload. Our study revealed the presence of a suite of synechobactins (seven of which are new variants), only in the extracts from the “zero-Iron (III) treatment” culture. 

## 2. Results and Discussion

### 2.1. Cultivation and Extraction of Anabaena flos-aquae UTEX 1444

**Figure 2:**
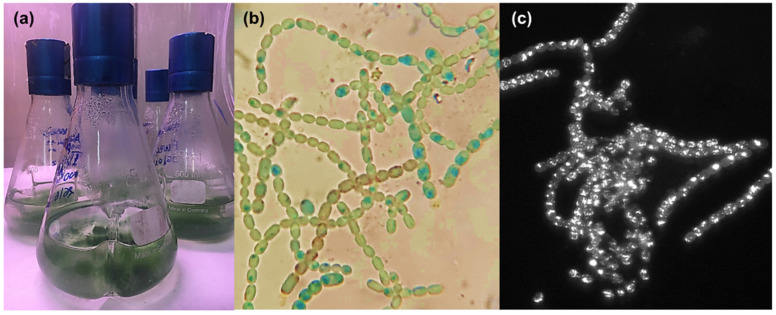
(**a**) Cultures of *Anabaena flos-aquae* UTEX 1444. Microscopic observation of 0 µM Fe^3+^ (#1) culture with (**b**) optical microscope and (**c**) fluorescence microscope (RFP filter).

The strain *Anabaena flos-aquae* UTEX 1444 (Figure 2, from the collection of Prof. A. Pollio) was grown in a BG11 culture medium [16], at six different Fe^3+^ concentrations, in the form of ferric ammonium citrate, yielding to #1–6 cultures: 0 µM Fe^3+^ (#1), 5 µM Fe^3+^ (#2), 10 µM Fe^3+^ (#3), 20 µM Fe^3+^ (#4), 60 µM Fe^3+^ (#5), and 100 µM Fe^3+^ (#6). Cultures #1–6 were extracted using our lab’s standard procedure [17]. Shortly, all cultures were vortexed, sonicated, and then centrifuged to separate the solid pellets from the liquid supernatant. Pellets were extracted with mixtures of MeOH and CHCl_3_ while supernatants were extracted with BuOH. All the extracts were then subjected to liquid chromatography coupled with high-resolution mass spectrometry (LC-HRMS). HRMS data were dereplicated using molecular networking analyses, as previously reported [18].

### 2.2. Molecular Networking and Synechobactins’ Identification

All extracts from cultures #1–6 were analyzed by LC-HRMS using an LTQ Orbitrap instrument. Data-dependent acquisition was used to trigger MS^2^ scans of the ten most intense ions detected in the full MS scan. The raw LC-MS data were pre-processed using the MZmine program 2.53 [19], which allowed us to remove isotopic peaks, to identify adducts and to perform quantitation. The preliminary analysis (data not shown) of MZmine data revealed that the most significant extract for each culture was that of butanol, as the most representative of each entire metabolome. Therefore, the subsequent data processing and molecular networking were performed on the butanol extracts, originating from #1–6 cultures. The global .mgf file, containing the MS^2^ data from #1–6 butanol extract and the quantitation table obtained by mzMine were submitted to the online platform at the Global Natural Products Social Molecular Networking website [20], where a Feature-Based Molecular Network (FBMN) was generated https://gnps.ucsd.edu/ProteoSAFe/status.jsp?task=664d019a6b52486d880014bb1e16bd69 (accessed on 12 November 2021) [21]. The resulting molecular network was then visualized using the Cytoscape program 3.9.0 [22] (see also Figures S1–S13, Supplementary Material).

**Figure 3:**
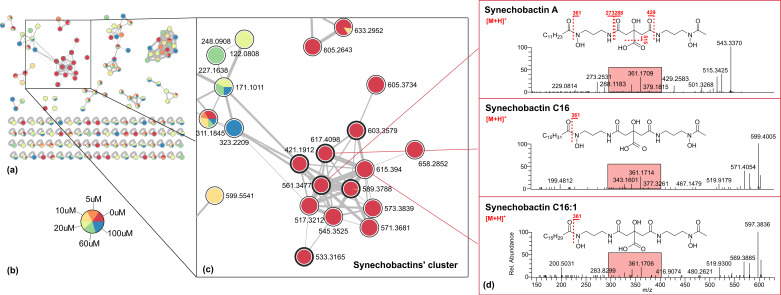
Molecular networking and HRMS/MS analysis of extracts of #1–6 cultures of *Anabaena flos-aquae* UTEX 1444. (**a**) The molecular network obtained combining the LC-HRMS/MS analyses of all the extracts of #1–6 cultures. Nodes are labeled with parent mass. (**b**) Nodes are represented as a pie chart showing the source culture of the compound ([Fe^3+^] = 0 µM (red), 5 µM (orange), 10 µM (dark yellow), 20 µM (yellow), 60 µM (green), 100 µM (blue)). (**c**) Cluster of synechobactins, present almost exclusively in zero iron (III) culture (0 µM, red). (**d**) HRMS/MS analysis and fragmentation pattern of synechobactin A, C_16_ and the new synechobactin C_16:1_.

The comprehensive quali-quantitative network of #1–6 butanol extracts (Figure 3a) contains 165 features, grouped into 10 clusters. In the network, each node is represented as a pie chart showing the amount of the compound in the source cultures containing different iron concentrations (Figure 3b).

One of the ten clusters was composed of 23 nodes, 13 of which were derived solely from the zero iron (III) culture (Figure 3c, nodes in red). These nodes did not match any known compounds in GNPS’ library. The ESI-HRMS spectrum of each of these compounds displayed, in addition to the [M + H]^+^ ion (*apo* form, not Fe-bound), an ion at 52.9117 amu difference [M + ^56^Fe − 3H]^+^, corresponding to their Fe-adduct, thus, suggesting the presence of Fe-chelating compounds. Six nodes were identified as synechobactins (Table 1), i.e., the hydroxamate-type siderophores. Synechobactins have a backbone consisting of citric acid, linked to two 1,3-diaminopropane units. The first diaminopropane unit is *N*-acetylated and *N*-hydroxylated, forming the hydroxamate group. The second diaminopropane unit is *N*-hydroxylated and *N*-acylated, with a fatty acid tail that varies in length and degree of unsaturation [23].

The first node of the series is schizokinen (*apo* [M + H]^+^, *m*/*z* 421.1934, C_16_H_29_O_9_N_4_^+^), the progenitor of the series, a symmetrical siderophore, in which both the two amino-terminals of the two diaminopropane units are acetylated, while the more lipophile synechobactin A (*apo* [M + H]^+^, *m*/*z* 561.3477, C_26_H_49_O_9_N_4_^+^) has a dodecanoyl chain. The ESI-HRMS^2^ spectrum of synechobactin A presents as a very peculiar fragment, due to the simultaneous loss of the acyl residue (C_12_H_22_O, 182.1661 amu) and a water molecule (probably originating from the citrate moiety), at *m*/*z* 361.1709 ([M – RCOH − H_2_O + H]^+^, C_14_H_25_O_7_N_4_^+^). This fragmentation is diagnostic, being present in all synechobactins [24], giving clear information about the nature of the molecule. Two additional fragments, originating from the sequential loss of water and the acetyl from the acetamide side (60.0206 amu, *m*/*z* [M + H]^+^ 501.3267, C_24_H_45_O_7_N_4_^+^) and of *N*-(3-aminopropyl)-*N*-hydroxyacetamide residue (132.0894 amu, *m*/*z* [M + H]^+^ 429.2582, C_21_H_37_O_7_N_2_^+^), were also observed, indicating the presence of the acetyl located on the second hydroxamate unit. Based on the typical fragmentation pattern of synechobactins, all the synechobactins of the cluster could be recognized. The ESI-HRMS^2^ spectra of synechobactin B (**3**), synechobactin C14 (**4**) and C16 (**5**), respectively, show the fragment ion at *m*/*z* 361.17 originated from the loss of the different fatty acid units, losses of 172.1465 amu (H_2_O and C_10_H_18_O), 228.2078 amu (H_2_O and C_14_H_26_O) and 256.2404 amu (H_2_O and C_16_H_30_O), respectively.

The molecular mass of **6**, corresponding to the molecular formula of C_30_H_55_O_9_N_4_^+^, was 2 amu (2 hydrogen atoms) less than that of synechobactin C_16_ (**5**), suggesting the presence of a double bond. The additional unsaturation was allocated to the fatty acid tail, the peak at *m*/*z* 361.1706 in the MS^2^ spectrum of **6** resulting from the neutral loss of the unsaturated fatty acid C_16_H_30_O_2_ (254.2235 amu). The position and the configuration of the double bond remained unassigned. To the best of our knowledge, the only reported synechobactin structure in which an unsaturated acyl chain is present is rhizobactin (C_10_) [25]; therefore, we propose that synechobactin C_16:1_ (**6**) is a new compound. Similarly, compound **7** has been identified as the new synechobactin oxyC_14_, having a hydroxy acyl (tetradecanoyl) chain, as indicated by the loss from the pseudomolecular ion of 244.2045 amu (H_2_O and C_14_H_26_O_2_). Compound **8** (*m*/*z* 603.3579 C_28_H_51_O_10_N_4_^+^) is also a derivative of synechobactin C_14_; specifically, the loss of 242.1879 amu (H_2_O and C_14_H_24_O_2_) yields the fragment ion at *m*/*z* 361.1702 (C_14_H_25_O_7_N_4_^+^), corresponding to the unsaturated hydroxytetradecanoic acid. The position and the configuration of the additional hydroxyl and the double bond remained unassigned.

Compounds (**1**–**8**) share the same dihydroxamate-citrate moiety, being different only in the structure of the fatty acid residue; compounds **9**–**12** are modified in the polar part. Both the cleavages from the pseudomolecular ions’ peaks at *m*/*z* 517.3212 (**9**) and 545.3525 (**10**) of dodecanoic and tetradecanoic acid, respectively, are observed for synechobactin A and C_14_, generating the fragment ion C_12_H_21_O_6_N_4_^+^ (*m*/*z* 317.1459), in consistence with the non-acetylated hydroxamate/citrate backbone. This was confirmed by the presence of the peak at *m*/*z* 429.2601, in the MS^2^ spectrum of **9,** due to the loss of the 3-(hydroxyamino)propan-1-amine (88.0634 amu, C_3_H_8_ON_2_) unit, which was also present in the MS^2^ spectrum of synechobactin A, due to the loss of the *N*-(3-aminopropyl)-*N*-hydroxyacetamide residue (132.0894 amu). The 15.9948 amu difference between synechobactin C_14_ (**4**) and the ion at *m*/*z* 573.3839 (compound **11**) accounts for an oxygen atom. The loss of tetradecanoic acid from the pseudomolecular ion of **11** leads to the peak at *m*/*z* 345.1772 (C_14_H_25_O_6_N_4_), indicating that the oxygen atom is missing in the dihydroxamate-citrate portion of the molecule. The peak at *m*/*z* 328.1508 (C_14_H_22_O_6_N_3_), in both the MS^2^ spectra of synechobactin C_14_ (**4**) and deoxyC_14_ (**11**), corresponds to the fragment (citryl)-*N*-(3-aminopropyl)-*N*-hydroxyacetamide, pointing to the lack of a hydroxyl group on the nitrogen of the other hydroxamate. Likewise, in compound **12**, the dehydrated form of deoxy synechobactin C_14_, dehydration occurs on the second hydroxamate unit. The thirteenth node of the synechobactins’ cluster (*m*/*z* 658.2852, C_28_H_50_O_10_N_4_Fe^+^) is the iron-bound ion of synechobactin oxyC_14_ (**7**).

## 3. Materials and Methods

### 3.1. Anabaena flos-aquae UTEX 1444: Strain, Cultivation and Extraction

The genus *Anabaena* BORY ex BORN. et FLAH. 1888 encompasses filamentous, non-branched species, which differ in the form of cells, coiling of trichomes, shape and size of akinetes [26]. *Anabaena* species exhibit a wide range of strain-specific variability, and environmental conditions mainly affect cell dimensions and size of heterocysts [27].

The species *A. flos-aquae* Brébisson ex Bornet et Flahault 1888, is a free-floating organism, whose coiled filaments can be solitary or irregularly assembled, with narrow mucilaginous sheath. Cells are usually spherical to barrel-shaped, whereas akinetes can be cylindrical or elliptical and heterocysts usually spherical [28]. *A. flos-aquae* is diffused in freshwater environments worldwide and is responsible of blooms that can have noxious effects on other organisms, due to the excretion of toxic metabolites, as anatoxins [29].

From a taxonomical point of view, *A. flos-aquae* is a combined morphological group, in that an alternation of regularly and irregularly coiled filaments is observed for the same strain in laboratory cultures; moreover, vegetative cell shape and size, along with heterocysts and akinetes dimensions, were largely variable among different populations [30]. Considering that the type species of the genus *Anabaena*, *A. oscillarioides* Bory ex Bornet et Flahault 1888 is a benthic type, Wacklin et al. [31] proposed to transfer the planktic *Anabaena* types under the genus *Dolichospermum*, designating *A. flos-aquae* as the type species of the genus.

The strain *Anabaena flos-aquae* UTEX 1444 was grown in BG-11 medium containing (per liter): 2.21 mg of Na_2_EDTA, 12 mg of Citric Acid, 1 g of NaNO_3_, 80 mg K_2_HPO_4_·3H_2_O, 150 mg of MgSO_4_·7H_2_O, 52.8 mg of CaCl_2_, 34.2 mg of NaCO_3_, 2 mL of Nitsch solution (H_2_SO_4_, MnSO_4_·H_2_O, ZnSO_4_·7H_2_O, CuSO_4_·5H_2_O, H_3_BO_3_, Na_2_MoO_4_·2H_2_O, CoCl_3_·6H_2_O), 500 µL of NiSO_4_ (NH_4_)_2_ SO_4_·6H_2_O (0.1 mM stock), 200 µL of Na_2_SeO_4_ (0.1 mM stock). Cultures were grown in six different concentrations of iron supplemented as ferric ammonium citrate in BG11 medium; serial dilutions from a mother ferric ammonium citrate solution (100 µM) were prepared in order to get the following final concentrations: 0 µM Fe^3+^ (#1), 5 µM Fe^3+^ (#2), 10 µM Fe^3+^ (#3), 20 µM Fe^3+^ (#4), 60 µM Fe^3+^ (#5), and 100 µM Fe^3+^ (#6). All cultures were made in triplicates and were incubated at room temperature (18–25 °C) for 30 days. Cultures were observed under an optical microscope (OPTECH, Biostar B3) and a fluorescence microscope (iRiS Digital Cell Imaging System–Logos Biosystems, Boston, MA, USA); then, they were vortexed, sonicated (ARGO Lab Digital Ultrasonic Cleaner, Model-DU 32, Carpi, Italy) at 20 KHz for 10 min at 23 °C, and centrifuged (HERMLE Labortechnik, Model-Z36HK, Wehingen, Germany) at 10,000 rpm for 5 min at 25 °C to separate the solid pellets from the liquid supernatant. Pellets were extracted with organic solvents as follows: MeOH (100%, 0.3 L), MeOH/CHCl_3_ (1:1, 0.3 L), and CHCl_3_ (100%, 0.3 L). All the extracts were paper filtered and concentrated under vacuum, yielding (average weights): 9.6 mg (#1), 6.6 mg (#2), 6.3 mg (#3), 9.0 mg (#4), 7.2 mg (#5) and 9.3 mg (#6) of MeOH extract; 2.3 mg (#1), 3.5 mg (#2), 4.3 mg (#3), 3.4 mg (#4), 1.2 mg (#5) and 2.5 mg (#6) of MeOH/CHCl_3_ extract; 7.6 mg (#1), 0.5 mg (#2), 1.7 mg (#3), 0.6 mg (#4), 1.3 mg (#5) and 0.3 mg (#6) of the CHCl_3_ extracts. Supernatants were extracted with BuOH. BuOH phases were concentrated under vacuum affording to 3.5 mg (#1), 13.3 mg (#2), 30.3 mg (#3), 45.6 mg (#4), 40.3 mg (#5) and 40.6 mg (#6) of extract. Each extract was resuspended in MeOH (100%, 5 mg/mL) for the subsequent LC-HRMS analyses.

### 3.2. LC-HRMS Analyses and Molecular Networking

LC-HRMS experiments were performed using a Thermo LTQ Orbitrap XL high-resolution ESI mass spectrometer (Thermo Fisher Scientific Spa, Rodano, Italy) coupled to an Agilent model 1100 LC system (Agilent Technologies, Cernusco sul Naviglio, Italy), which included a solvent reservoir, in-line degasser, binary pump, and refrigerated autosampler. A 5-µm Kinetex C18 column (50 × 2.10 mm), maintained at room temperature, was eluted at 200 mL min^−1^ with H_2_O (supplemented with 0.1% HCOOH) and MeOH, using gradient elution. The gradient program was as follows: 10% MeOH for 3 min, 10%→100% MeOH for 30 min, 100% MeOH for 7 min. Mass spectra were acquired in positive ion detection mode. MS parameters were a spray voltage of 4.8 kV, a capillary temperature of 285 °C, a sheath gas rate of 32 units N_2_ (ca. 150 mL/min), and an auxiliary gas rate of 15 units N_2_ (ca. 50 mL/min). Data were collected in the data-dependent acquisition (DDA) mode, in which the first, the second, up until the tenth most intense ions of a full-scan mass spectrum were subjected to high-resolution tandem mass spectrometry (HRMS/MS) analysis. The *m*/*z* range for data-dependent acquisition was set between 100 and 2000 amu. HRMS/MS scans were obtained for selected ions with CID fragmentation, isolation width of 2.0, normalized collision energy of 35, Activation Q of 0.250, and activation time of 30 ms. Data were analyzed using Thermo Xcalibur software.

Raw files were imported into MZmine 2.53 open-source software [14]. The mass detection was performed on raw data and exact masses with mass level 1 and centroided masses with mass level 2, by keeping the noise level at 1000. Chromatograms were built using an ADAP module with a minimum height of 1000, and *m*/*z* tolerance of 0.001 (or 5 ppm). Peak alignment was performed using the Join aligner algorithm (*m*/*z* tolerance at 0.005 (or 5 ppm), absolute RT tolerance at 0.2 min). [M + Na – H], [M + K – H], [M + Mg − 2H], [M + NH_3_], [M − Na + NH_4_], [M + 1, ^13^C], [M − ^35^Cl + ^37^Cl]^+^, [M + ^56^Fe − 3H]^+^ adducts were filtered out by setting the maximum relative height at 100%. Peaks without associated MS/MS spectra were finally filtered out from the peak list. Clustered data were then exported to .mgf file for GNPS, while chromatographic data including retention times, peak areas, and peak heights were exported to a .csv file.

A Feature-Based Molecular Network was generated on GNPS’ online platform [15], with the following parameters: the parent mass tolerance and MS/MS fragment ion tolerance were set both at 0.05 Da, the cosine score at above 0.5, and matched peaks above 5. Spectra were retained only if the nodes appeared in each other’s respective top 10 most similar nodes. The spectra in the network were then searched against GNPS spectral libraries using a cosine score above 0.7 and at least 6 matched peaks. The molecular network was visualized using Cytoscape software [16].

## 4. Conclusions

It is well known that cyanobacteria have a unique adaptive character due to their metabolism capability to react to adverse environmental conditions. They represent a large group of bioagents, less explored as bioresources that produce high-value natural products with biotechnological and ecological relevance. This paper reports on our study on the cyanobacteria *Anabaena flos-aquae* strain UTEX 1444, as a promising sustainable bioresource of bioactive molecules, i.e., siderophores. We explored metabolome variations when UTEX 1444 was cultivated in the condition of iron deficiency and iron overload. As presumed, the cultivation of the strain in conditions of iron deficiency revealed its capability to produce siderophores. In our procedure, to obtain a fast dereplication of #1–6 organic extracts, molecular networking analyses of MS/MS data were successfully used. Comprehensive FBMN, featuring #1–6 metabolites’ relative abundance comparison, immediately disclosed the presence of a cluster composed of metabolites, belonging only to the zero iron (III) culture and, therefore, allowed us to easily and quickly pinpoint the siderophore cluster; moreover, molecular networking allowed for a fast detection of synechobactins and new variants, thanks to their typical fragmentation pattern. HRMS/MS analysis and the fragmentation pattern of synechobactin A, C_16_ and the new synechobactin C_16:1_ are reported as a useful diagnostic way to detect them in any extract. The structure and the kind of cyanobacterial siderophores were determined in only a few previously published papers [7].

Together with the five known synechobactins, six new variants have been identified, showing unusual modifications occurring in the nature of the acyl chain (unsaturated, hydroxylated) and in the hydroxamate-citrate backbone. To our best knowledge, this is the first report unveiling the biosynthetic capability of UTEX 1444 to produce a large array of synechobactins. It is interesting to note that UTEX 1444 produced 11 variants, beside schizokinen, showing the ability to incorporate a variety of saturated and unsaturated fatty acids, likely as a final biosynthetic step. Modifications also occur in the hydroxamate-citrate backbone, extending the chemical variants of the synechobactins known so far.

Considering the potential high impact of siderophores production as ferric ion scavengers, as well as toxic metal chelators, and then, the large array of applications in many different fields, from ecological research to drug discovery [5,6,7], our future studies will be focused on making UTEX 1444 a suitable model organism for a scale-up production of siderophores. This report, encompassing methodological improvements in siderophore production, characterization and detection through the dereplication of extracts, using a powerful bioinformatic approach, is a sustainable contribution to quest natural molecules that improve environmental and human health. Following EU direction, this study follows the “Do no significant harm” requirement and overcomes the “supply” problem that is the main bottleneck in the study of novel bioactive molecules from marine organisms.

## Figures and Tables

**Table 1:** Synechobactins in *Anabaena flos-aquae* UTEX 1444. The new variants are highlighted in yellow.

No.	Name	*apo* [M + H]^+^	Molecular Formula	t_R_ (min)
**1**	Skizokinen	421.1934	C_16_H_29_O_9_N_4_^+^	4.7
**2**	Synechobactin A	561.3477	C_26_H_49_O_9_N_4_^+^	30.7
**3**	Synechobactin B	533.3165	C_24_H_45_O_9_N_4_^+^	27.7
**4**	Synechobactin C_14_	589.3788	C_28_H_53_O_9_N_4_^+^	33.0
**5**	Synechobactin C_16_	617.4098	C_30_H_57_O_9_N_4_^+^	35.1
**6**	Synechobactin C_16:1_	615.3940	C_30_H_55_O_9_N_4_^+^	33.6
**7**	Synechobactin oxyC_14_	605.3734	C_28_H_53_O_10_N_4_^+^	30.9
**8**	Synechobactin oxyC_14:1_	603.3579	C_28_H_51_O_10_N_4_^+^	30.7
**9**	Desacetyl-synechobactin A	517.3212	C_24_H_45_O_8_N_4_^+^	30.3
**10**	Desacetyl-synechobactin C_14_	545.3525	C_26_H_49_O_8_N_4_^+^	32.7
**11**	Deoxysynechobactin C_14_	573.3839	C_28_H_53_O_8_N_4_^+^	32.4
**12**	Deoxysynechobactin C_14:1_	571.3681	C_28_H_51_O_8_N_4_^+^	33.3

## Supplementary Materials

The following are available online at https://www.mdpi.com/article/10.3390/md20040256/s1, Figure S1: Global molecular network of *A. flos-aquae* UTEX 1444’s extracts, Figure S2: MS/MS spectrum of schizokinen (**1**), Figure S3: MS/MS spectrum of synechobactin A (**2**), Figure S4: MS/MS spectrum of synechobactin B (**3**), Figure S5: MS/MS spectrum of synechobactin C_14_ (**4**), Figure S6: MS/MS spectrum of synechobactin C_16_ (**5**), Figure S7: MS/MS spectrum of putative synechobactin C_16:1_ (**6**), Figure S8: MS/MS spectrum of putative synechobactin C_14_OH (**7**), Figure S9: MS/MS spectrum of putative synechobactin deacA (**8**), Figure S10: MS/MS spectrum of putative synechobactin deacC_14_ (**9**), Figure S11: MS/MS spectrum of putative synechobactin deoxyC_14_ (**10**), Figure S12: MS/MS spectrum of putative synechobactin dehydroC_14_ (**11**), Figure S13: MS/MS spectrum of putative synechobactin C_14:2_OH (**12**).
