# Supplementary material for: A Glimpse at Siderophores Production by Anabaena flos-aquae UTEX 1444
Source: Mar Drugs. 2022 Apr 6;20(4):256. doi: 10.3390/md20040256 (PMC9025534; doi:10.3390/md20040256)
Supplement: Supplementary file 1 [file marinedrugs-20-00256-s001.zip › marinedrugs-1627456-supplementary.pdf]

Supplementary material

A Glimpse at siderophores production by *Anabaena Flos-aquae* UTEX 1444

Roberta Teta [1], Germana Esposito [1], Karishma Kundu[1], Mariano Stornaiuolo[2], Silvia Scarpato[1], Antonino Pollio[3], and Valeria Costantino [1]*

[1] "TheBlueChemistryLab", Department of Pharmacy, Task force "BIGFED2", University of Naples Federico II, via Domenico Montesano 49, 80131 Napoli, Italy; roberta.teta@unina.it (R.T.), germana.esposito@unina.it (G.E.), karishma.kundu@unina.it (K.K), mariano.stornaiuolo@unina.it (M.S.), silvia.scarpato@unina.it (S.S.), valeria.costantino@unina.it (V.C.)

[2] Department of Pharmacy, University of Naples Federico II, via Domenico Montesano 49, 80131 Napoli, Italy; mariano.stornaiuolo@unina.it (M.S.)

[3] Department of Biology, University of Naples Federico II, Complesso Universitario Monte Sant'Angelo via Cinthia – Edificio 7, 80126 Napoli, Italy; antonino.pollio@unina.it (A.P.)

* Correspondence: valeria.costantino@unina.it (V.C.)

Table of contents

Figure S1. Global molecular network of *A. flos-aquae* UTEX 1444's extracts .... p. 4

Figure S2. MS/MS spectrum of schizokinen (**1**) .... p. 5

Figure S3. MS/MS spectrum of synechobactin A (**2**) .... p. 4

Figure S4. MS/MS spectrum of synechobactin B (**3**) .... p. 4

Figure S5. MS/MS spectrum of synechobactin C_{14} (**4**) .... p. 4

Figure S6. MS/MS spectrum of synechobactin C_{16} (**5**) .... p. 4

Figure S7. MS/MS spectrum of putative synechobactin $C_{16:1}$ (**6**) .... p. 4

Figure S8. MS/MS spectrum of putative synechobactin oxyC_{14} (**7**) .... p. 4

Figure S9. MS/MS spectrum of putative synechobactin oxy$C_{14:1}$ (**8**) .... p. 4

Figure S10. MS/MS spectrum of putative desacetyl-synechobactin A (**9**) .... p. 4

Figure S11. MS/MS spectrum of putative desacetyl-synechobactin C_{14} (**10**) .... p. 4

Figure S12. MS/MS spectrum of putative deoxysynechobactin C_{14} (**11**) .... p. 4

Figure S13. MS/MS spectrum of putative deoxysynechobactin $C_{14:1}$ (**12**) .... p. 4

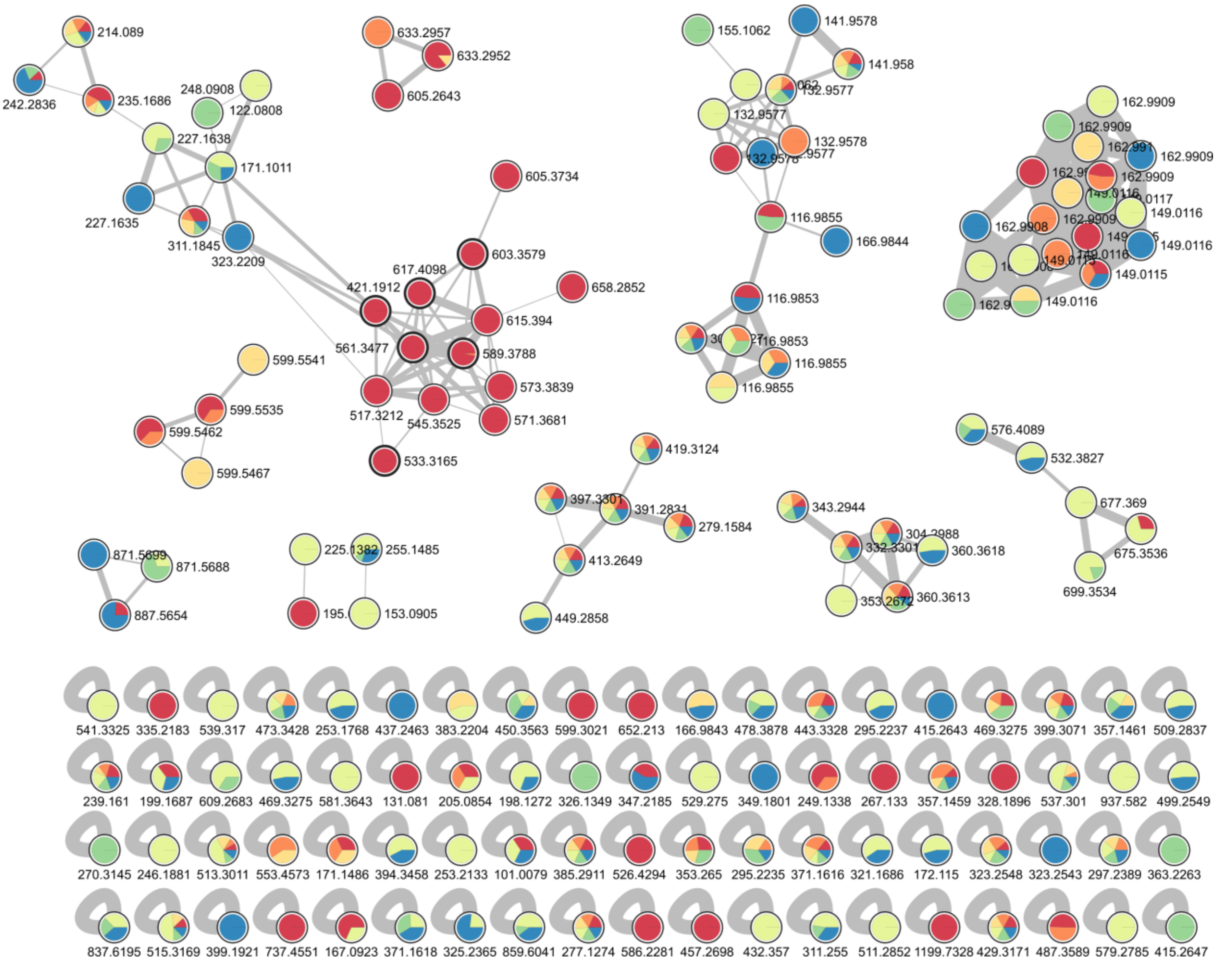

Figure S1. Global molecular network of *A. flos-aquae* UTEX 1444's extracts

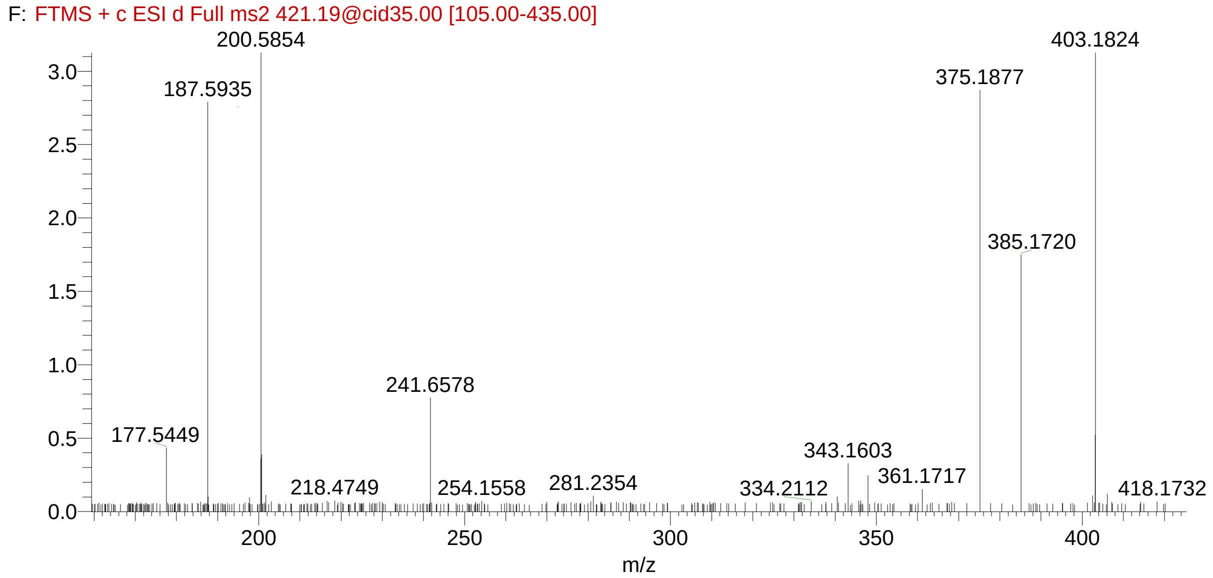

Figure S2. MS/MS spectrum of schizokinen (**1**)

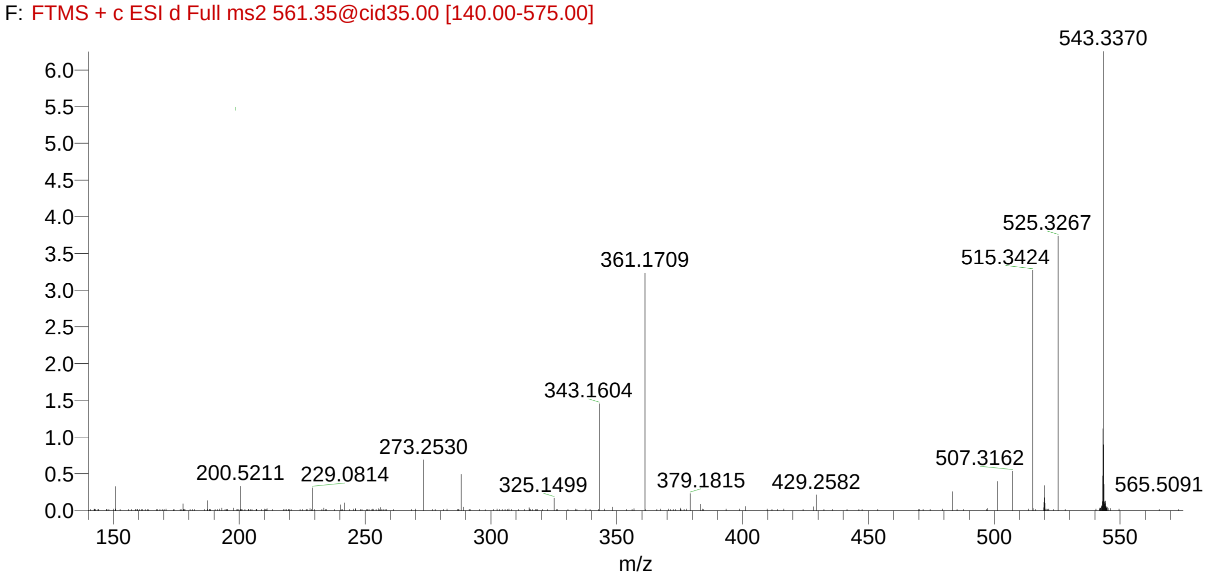

Figure S3. MS/MS spectrum of synechobactin A (**2**)

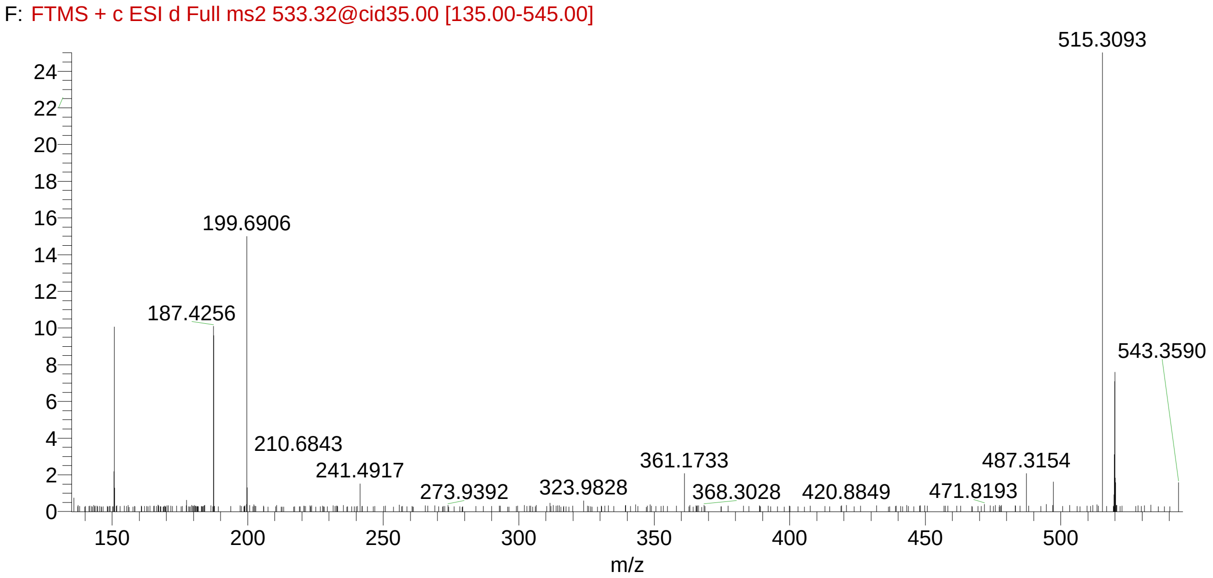

Figure S4. MS/MS spectrum of synechobactin B (**3**)

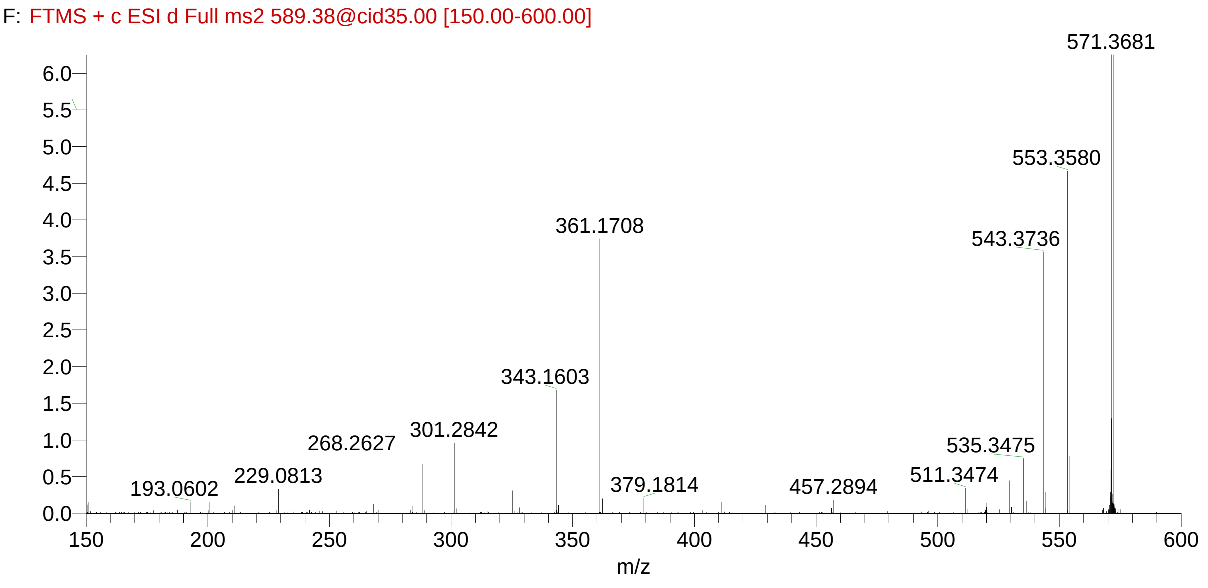

Figure S5. MS/MS spectrum of synechobactin C_{14} (**4**)

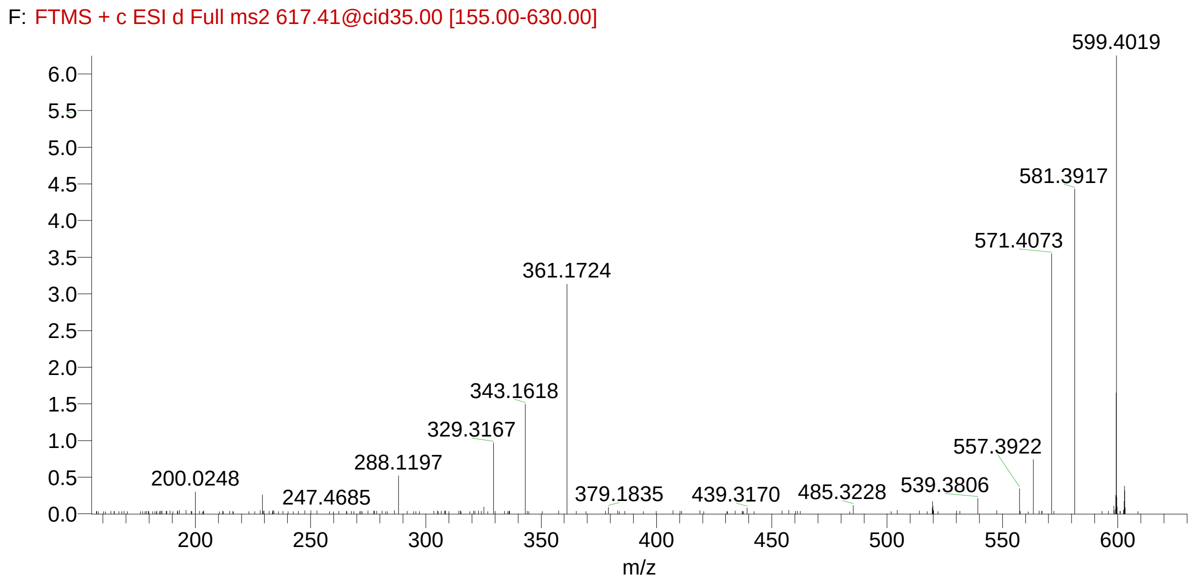

Figure S6. MS/MS spectrum of synechobactin C_{16} (**5**)

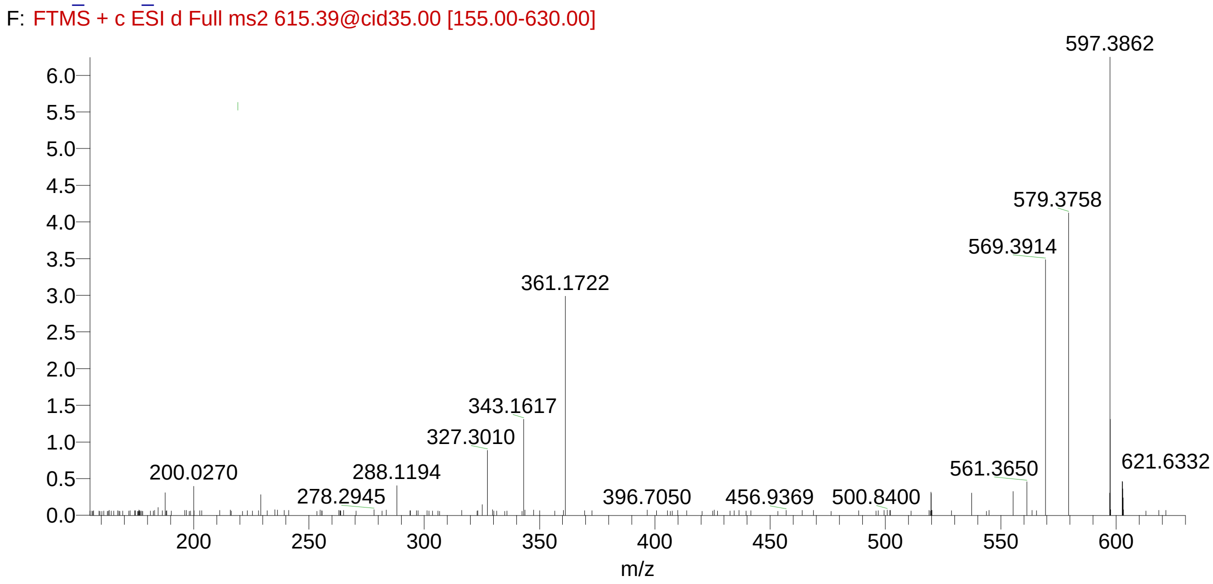

Figure S7. MS/MS spectrum of putative synechobactin $C_{16:1}$ (**6**)

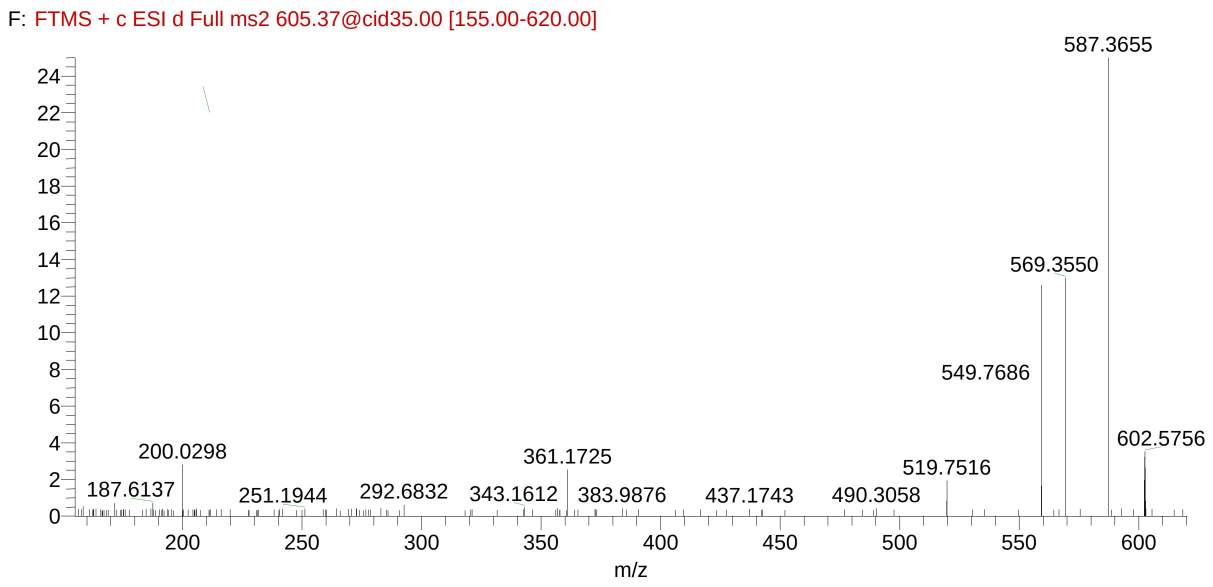

Figure S8. MS/MS spectrum of putative synechobactin oxyC_{14} (**7**)

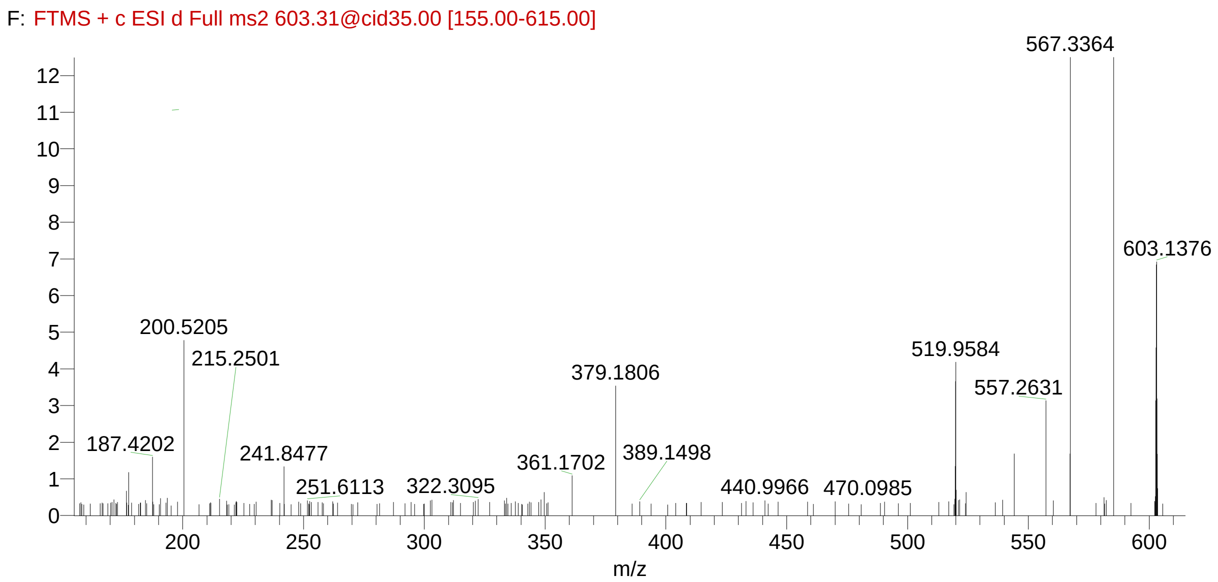

Figure S9. MS/MS spectrum of putative synechobactin oxy$C_{14:1}$ (**8**)

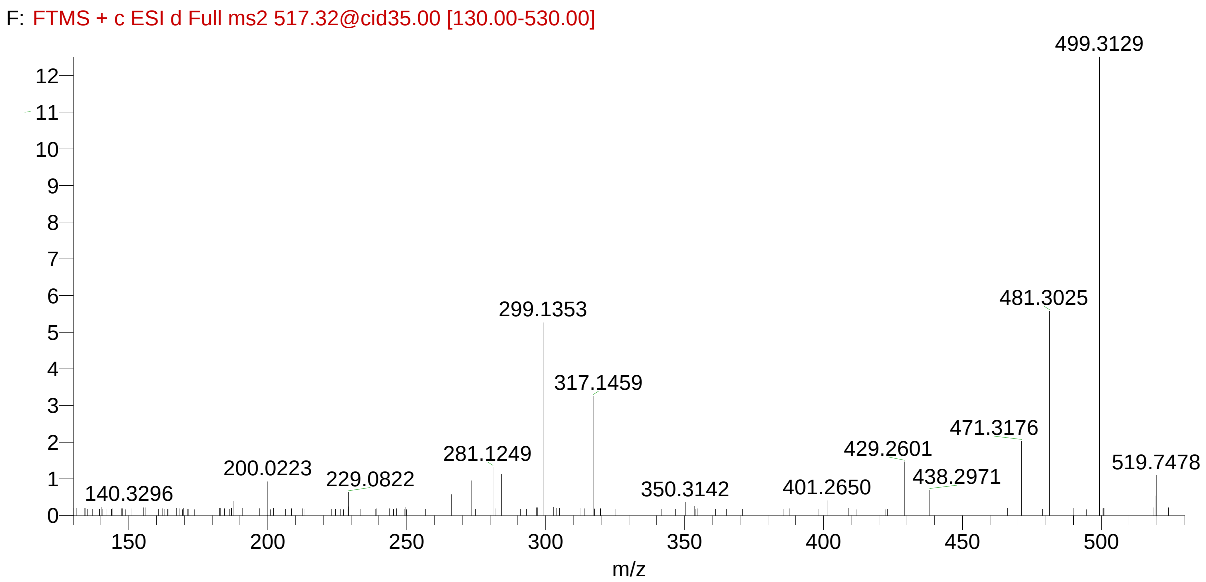

Figure S10. MS/MS spectrum of putative desacetyl-synechobactin A (**9**)

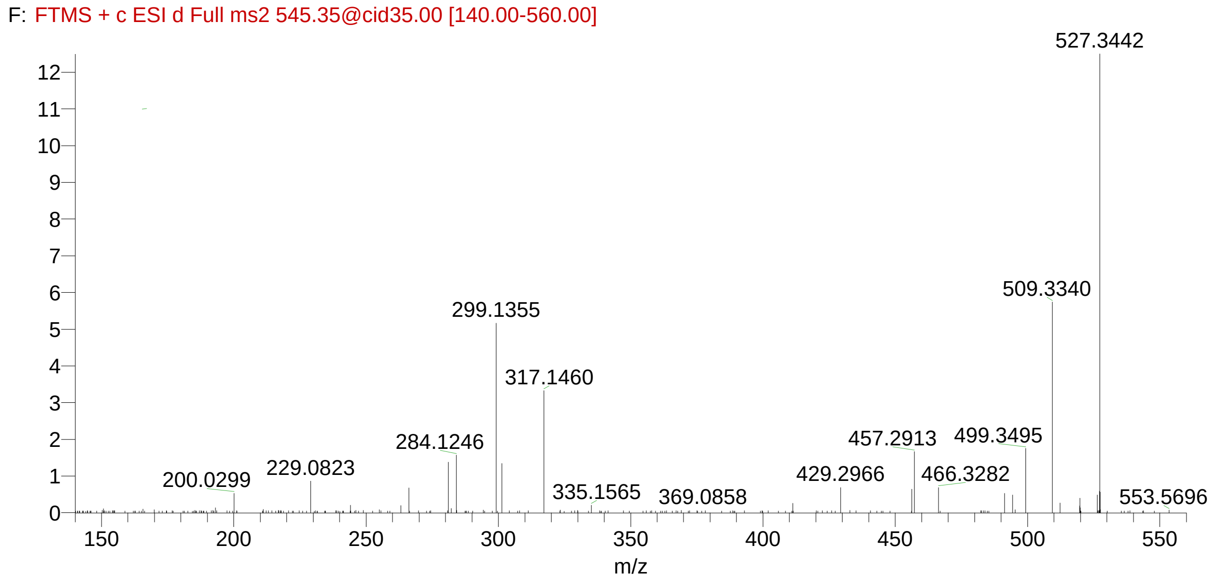

Figure S11. MS/MS spectrum of putative desacetyl-synechobactin C_{14} (**10**)

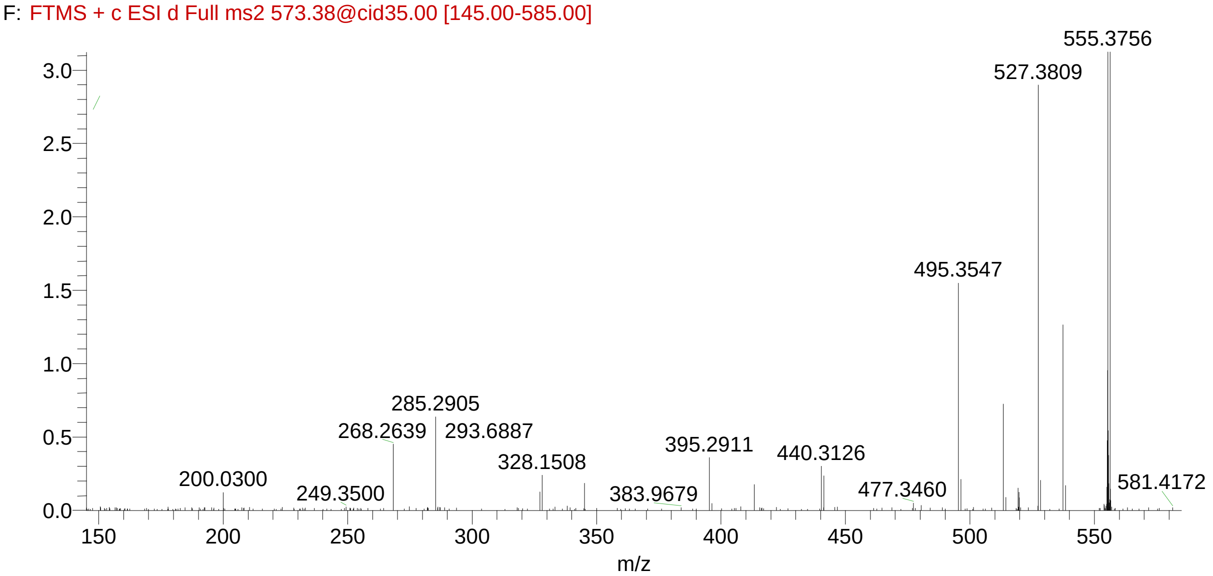

Figure S12. MS/MS spectrum of putative deoxysynechobactin C_{14} (**11**)

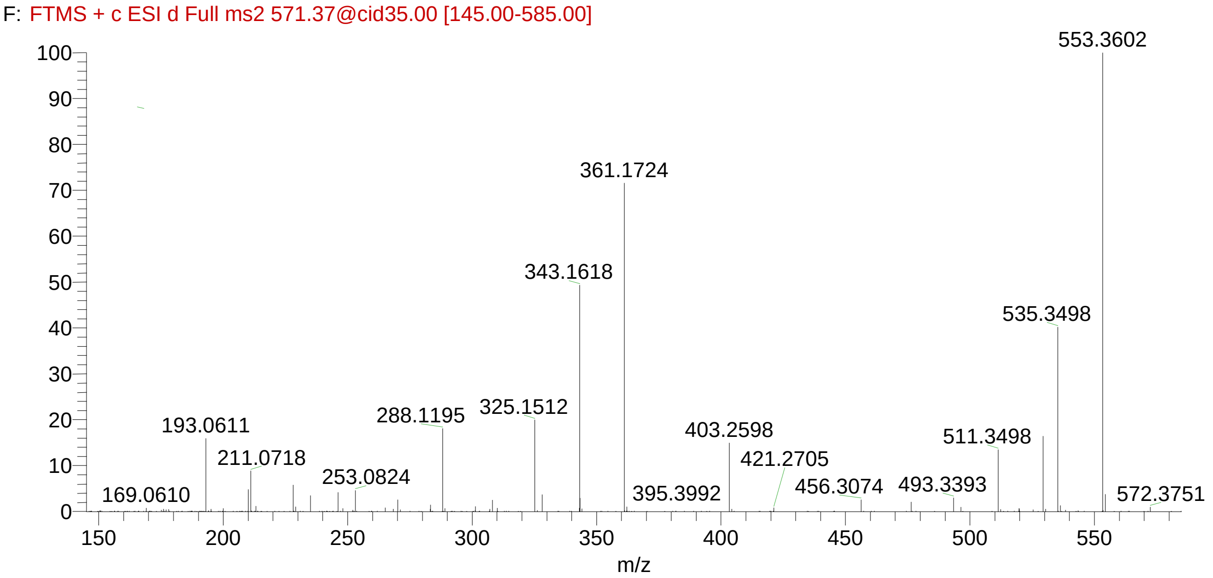

Figure S13. MS/MS spectrum of putative deoxysynechobactin $C_{14:1}$ (**12**)
